# *Akkermansia muciniphila* ameliorates olanzapine-induced metabolic dysfunction-associated steatotic liver disease via PGRMC1/SIRT1/FOXO1 signaling pathway

**DOI:** 10.3389/fphar.2025.1550015

**Published:** 2025-03-19

**Authors:** Hui Chen, Ting Cao, ChenQuan Lin, ShiMeng Jiao, YiFang He, ZhenYu Zhu, QiuJin Guo, RenRong Wu, HuaLin Cai, BiKui Zhang

**Affiliations:** ^1^ Department of Pharmacy, Changsha Stomatological Hospital, Changsha, Hunan, China; ^2^ Department of pharmacy, Institute of Clinical Pharmacy, The Second Xiangya Hospital of Central South University, Changsha, Hunan, China; ^3^Department of Psychiatry, National Clinical Research Center for Mental Disorders, The Second Xiangya Hospital of Central South University, Changsha, Hunan, China; ^4^ Xiangya School of Pharmaceutical Sciences, Central South University, Changsha, Hunan, China; ^5^ International Research Center for Precision Medicine, Transformative Technology and Software Services, Changsha, Hunan, China

**Keywords:** AKK, OLZ, MASLD, lipid accumulation, insulin resistance, PGRMC1

## Abstract

*Akkermansia muciniphila* (AKK), classified as “lean bacteria,” has emerged as a promising candidate for ameliorating metabolic disorders, including obesity, diabetes, and liver disease. In this study, we investigated the therapeutic potential of AKK to counteract metabolic dysfunctions induced by Olanzapine (OLZ), a first-class antipsychotic known for its high therapeutic efficacy but also its association with metabolic disturbances, particularly Metabolic Dysfunction-Associated Steatotic Liver Disease (MASLD). Previous studies have implicated progesterone receptor membrane component 1 (PGRMC1) as a key player in antipsychotic-induced metabolic side effects. Using male C57BL/6J mice fed a high-fat diet, we assessed the effects of AKK supplementation on OLZ-induced metabolic disturbances. Key parameters such as body weight, hepatic injury markers, glucose tolerance, insulin resistance, and lipid metabolism were analyzed. The study revealed that AKK supplementation reduced hepatic lipid accumulation, oxidative stress, and insulin resistance, while normalizing lipid and glucose metabolism. These effects are likely mediated through the restoration of PGRMC1/SIRT1/FOXO1 signaling pathway by AKK. Additionally, changes in gut microbiota composition, including a reduction in pathogenic bacteria such as *Lactococcus* and enrichment of beneficial bacteria, were observed. Overall, the study suggests that AKK has therapeutic potential to counteract OLZ-induced MASLD by modulating gut microbiota and key metabolic pathways, making it a promising strategy for managing metabolic side effects in patients receiving antipsychotic treatment.

## 1 Introduction

Schizophrenia (SZ) is a chronic severe psychiatric disorder impacting cognition, perception, and behavior, with a global prevalence of approximately 1% (Charlson et al., 2018; Kanchanatawan et al., 2018). Olanzapine (OLZ), a first-line antipsychotic, is valued for its high therapeutic efficacy and low incidence of extrapyramidal side effects (Leucht et al., 2013). However, long-term OLZ use in patients with SZ is associated with an increased risk of metabolic side effects, particularly Metabolic Dysfunction-Associated Steatotic liver disease (MASLD) (Hert et al., 2012; Olfson et al., 2015; Marder and Cannon, 2019). The term MASLD was introduced to better describe the spectrum of fatty liver diseases associated with systematic metabolic dysfunction (Gofton et al., 2023; Rinella et al., 2023). In our previous study (Zhu et al., 2023), we found that OLZ treatment alone or combined with either a high-fat or normal diet could induce MASLD in C57BL/6 mice. However, the combination of OLZ and a high-fat diet significantly aggravated liver injury and oxidative stress, suggesting that a high-fat diet exacerbates the metabolic disturbances induced by OLZ. This finding underscores the high-fat diet as a critical risk factor for MASLD development in patients with SZ undergoing antipsychotic treatment. Notably, clinical observations found that SZ patients preferred a high-fat diet, which may further exacerbate metabolic side effects (Dipasquale et al., 2013). Therefore, we used OLZ combined with a high-fat diet to develop the MASLD model in our study.

Emerging evidence underscores the pivotal role of gut microbiota in the pathogenesis of MASLD (Le Roy et al., 2013; Albillos et al., 2020; Song and Zhang, 2022; Chen et al., 2023). Notably, both clinical and animal studies have demonstrated that OLZ administration altered the composition of gut microbiota, potentially exacerbating metabolic disorders (Morgan et al., 2014; Kao et al., 2018; Pełka-Wysiecka et al., 2019; Zeng et al., 2024). Specifically, OLZ reduced the abundance of *Akkermansia muciniphila* (AKK) in rodents (Zhu et al., 2022; Zeng et al., 2024). Intriguingly, decreased AKK abundance is also observed in both patients with MASLD (Oh et al., 2021) and mouse models (Rao et al., 2021; Wu et al., 2023), mirroring the effects of OLZ. This congruence suggests a potential role for AKK in OLZ-induced MASLD. Furthermore, AKK has been shown to exhibit protective effects against MASLD induced by a high-fat diet, including lipid-lowering, anti-inflammatory, immunomodulatory, and insulin-sensitizing properties (Maestri et al., 2023). However, the precise mechanisms underlying AKK*’*s involvement in OLZ-induced MASLD remain to be fully elucidated.

AKK is a promising gut bacterium known for its beneficial effects in mitigating metabolic disorders through several mechanisms, including the enhancement of mitochondrial oxidative function, modulation of bile acid metabolism, and reduction of oxidative stress-induced apoptosis in the gut (Rao et al., 2021). These mechanisms not only contribute to reshaping the gut microbiota composition but also improve metabolic conditions in the liver. Notably, AKK modulates the gut-liver axis by regulating the intestinal FXR-FGF15 signaling pathway and altering bile acid profiles, thereby reducing levels of secondary bile acids in the cecum and liver (Wu et al., 2023). Certain studies have further demonstrated the role of prebiotics, such as B-GOS (a type of bifidogenic oligosaccharide), in enhancing the abundance of AKK in the gut. This increase in AKK has been associated with a significant improvement in OLZ-induced gut microbiota imbalance and hepatic lipid dysregulation in mice. Additionally, these studies revealed that the beneficial effects of AKK are linked to the activation of the hepatic progesterone receptor membrane component 1 (PGRMC1)-related pathway (Zeng et al., 2024). This makes PGRMC1 a potential therapeutic target for addressing metabolic side effects. However, the exact mechanisms by which AKK influences PGRMC1 expression and function, particularly in the context of antipsychotic-induced metabolic dysregulation, remain largely unexplored.

PGRMC1 is a multifunctional protein that plays a key role in various intracellular processes, including metabolism, signal transduction, and steroid signaling (Cahill and Neubauer, 2021). It modulates intracellular lipid and energy homeostasis by regulating lipid synthesis and degradation (Cai et al., 2015; Lee et al., 2018). Its potential role in mitigating the metabolic side effects of antipsychotic drugs, including OLZ and clozapine, has garnered increasing interest. Notably, PGRMC1 exhibits high affinity for antipsychotic drugs (Rohe et al., 2009) such as olanzapine (Zhu et al., 2023) and clozapine (Cao et al., 2021). Both *in vivo* and *in vitro* evidence has demonstrated that knockdown or overexpression of hepatic PGRMC1 can mimic or mitigate the metabolic effects induced by antipsychotic drugs, including lipid accumulation and impaired glucose metabolism (Lee et al., 2018; Cao et al., 2021; Zhu et al., 2023). This suggests that PGRMC1 appears to act as a critical mediator, connecting antipsychotic-induced metabolic disturbances with gut microbial modulation.

As previously reported, overexpression of hepatic PGRMC1 in rats ameliorated hepatic glucose metabolism disorders induced by clozapine, along with the downregulation of nuclear FOXO1 (Cao et al., 2021). FOXO1, a member of the FOXO family, is a transcription factor that plays a central role in insulin signaling and lipid metabolism by regulating genes involved in gluconeogenesis and lipid metabolism (Lee and Dong, 2017; Lees et al., 2023). Under oxidative stress conditions, FOXO1 can induce the expression of genes promoting hepatic lipid accumulation, potentially exacerbating MASLD (Tikhanovich et al., 2013). Given the similarities between OLZ and clozapine in chemical structure and receptor binding properties, we speculate that the metabolic disorders induced by OLZ may also be mediated through the PGRMC1-FOXO1 signaling pathway.

Beyond intracellular signaling, PGRMC1 influences lipid metabolism through the key deacetylase, silent information regulator 1 (SIRT1), thereby affecting overall metabolic homeostasis (You et al., 2021). In the liver, SIRT1 enhances fatty acid oxidation while inhibiting synthesis, crucial for preventing fat accumulation and MASLD progression (Ryall et al., 2015; Wątroba et al., 2023). A connection between FOXO1 and SIRT1 has also been proposed (Sasaki and Kitamura, 2010). SIRT1 modulates FOXO1 activity through deacetylation, thereby enhancing FOXO1’s nuclear retention and sustaining the downstream signaling pathway mediated by FOXO1 (Qiang et al., 2010; Sin et al., 2015). Hence, it is likely that PGRMC1 interfaces with metabolic regulation via the SIRT1/FOXO1 axis, contributing to the progression of MASLD.

We hypothesize that AKK has therapeutic effects in treating MASLD induced by OLZ combined with HFD, which is known to exacerbate hepatocyte injury and drive MASLD progression. Additionally, we propose that the hepatic PGRMC1/SIRT1/FOXO1 signaling pathway plays a key role in this process. By investigating these mechanisms, we aim to better understand the pathological underpinnings of OLZ-induced MASLD and identify potential therapeutic strategies.

## 2 Materials and methods

### 2.1 Animals

To avoid estrogen fluctuations on hepatic PGRMC1 expression, male mice were selected in our study. The male C57BL/6J mice (7–8 weeks, 20 ± 1.0 g) were purchased from Slack Jingda Experimental Animal Co., Ltd (Changsha, China) and housed at the Experimental Animal Department of Central South University. Mice were kept under a 12-hour light-dark cycle at 24°C–26°C with 50% ± 10% humidity and had ad libitum access to food and water. The study was approved by the Laboratory Animal Welfare Ethics Committee of Central South University, following the Administrative Regulations on Laboratory Animals (Ethics approval number: CSU-2022-0456).

### 2.2 Experimental design

**FIGURE 1:**
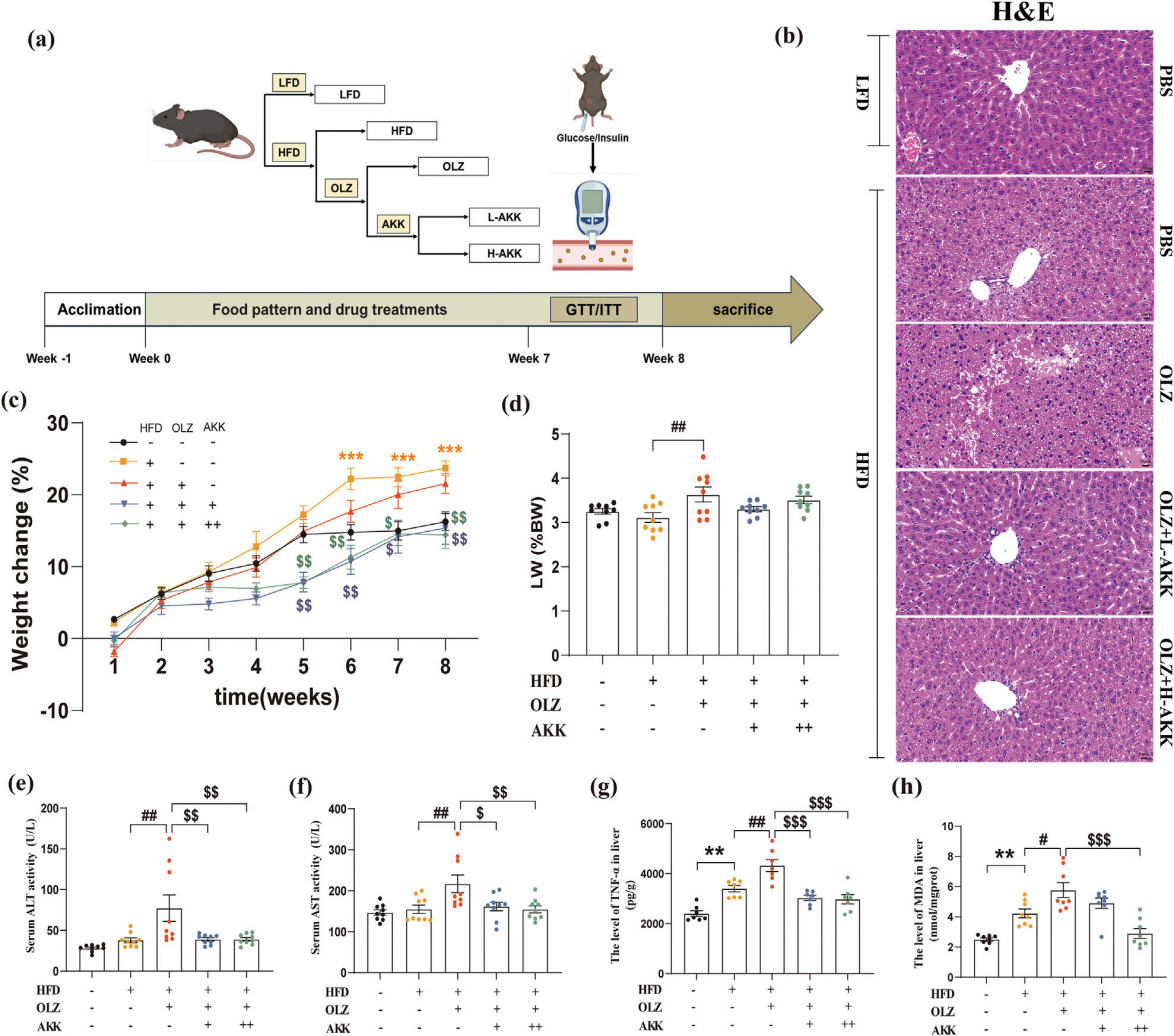
Effects of AKK on olanzapine-induced weight gain and hepatic injury. **(a)** A diagram of experimental design and the time course illustrating when each procedure took place. **(b)** H&E staining (scale bar = 20 um). **(c)** The percent of body weight gain (%). **(d)** The ratio of liver weight to body weight. **(e)** Serum ALT activity. **(f)** Serum AST activity. **(g)** The level of TNF-α in liver. **(h)** The levels of MDA in liver. Differences were considered statistically significant when *p* < 0.05. ^*^ *p* < 0.05, ^**^ *p* < 0.01, ^***^ *p* < 0.001, HFD vs. LFD. ^#^ *p* < 0.05, ^##^ *p* < 0.01, ^###^ *p* < 0.001, OLZ vs. HFD. ^$^ *p* < 0.05, ^$$^ *p* < 0.01, ^$$$^ *p* < 0.001, OLZ-AKK vs. OLZ-PBS.

45 male C57BL/6J mice were randomized into 5 experimental groups based on body weight (n = 9/group). After a week of acclimatation, drugs were administered as Figure 1a. The dosage of OLZ (Macklin, China) was set at 20 mg/kg/day via intraperitoneal injection to mimic a high clinical dose (Kane et al., 2003; Petersen et al., 2014) and long-term administration of this dose was successful in inducing metabolic dysfunction in mice (Zhu et al., 2023). Additionally, mice in the AKK intervention group were orally gavaged with 200 μL/day AKK capsule, containing 1 × 10^8^ active fluorescent units (AFU) of AKKWB-STR-0001 (Pendulum, United States) (L-AKK: 1×10^7^ AFU/kg; H-AKK: 2×10^7^ AFU/kg). The low dose of AKK was equivalent to the recommended dose of commercial AKK products. Body weight and food intake were monitored. After 8 weeks, feces were collected. Mice were then fasted for 12 h, anesthetized intraperitoneally, and blood samples collected from eye sockets. A portion of the liver was fixed for histological analysis, while the rest of the liver and cecum contents were frozen in liquid nitrogen and stored at −80°C.

### 2.3 Glucose and insulin tolerance analyses

On the first day of the eighth week post-administration, a glucose tolerance test (GTT) was conducted. Following a 16-hour fast, mice were intraperitoneally injected with a 20% glucose solution at a dosage of 2 g/kg. Blood glucose levels in the tail vein were measured at 15, 30, 60, 90, and 120 min post-injection using a glucometer (Sinocare, China). Three days after the GTT, an insulin tolerance test (ITT) was performed. Prior to the experiment, all mice underwent a 4-hour fast, followed by intraperitoneal injection of an insulin solution at a dosage of 0.75 IU/kg. Blood glucose levels in the tail vein were measured at 15, 30, 60, 90, and 120 min post-injection. Blood glucose curves were plotted for each group of mice, and the area under the curve (AUC) was calculated.

### 2.4 Biochemical analyses

Fasting blood glucose was measured by a glucometer (Sinocare, China) in mice. Additionally, ALT, AST, TC, TG, and NEFA levels in serum and liver tissues were measured by a fully automated biochemical analyzer (Biobase, China). Serum levels of insulin (Jingmei Biotechnology, AF2579-A) and glucagon (Jingmei Biotechnology, AF2167-A), as well as liver tissue levels of TNF-α (Jingmei Biotechnology, AF2132-A) and MDA (Nanjing Jiancheng Biotechnology Research Institute, A003-1), were measured using ELISA kits.

### 2.5 Histological analysis

Hematoxylin-eosin (H&E) staining: Liver tissue was removed from the fixative, dehydrated in ethanol, infiltrated with paraffin, and embedded. The tissue was then sectioned (4 μm) and deparaffinized, followed by staining with hematoxylin and eosin, dehydration, and sealing with neutral gum. Microscopic examination and image acquisition were carried out afterwards.

Oil Red O staining: Fixed tissues were dehydrated in a sucrose solution and rapidly embedded. Sections (8–10 μm) were cut and mounted onto slides. These sections were then stained with the Oil Red O solution (covered to avoid light exposure), differentiated in 60% isopropanol. Subsequently, the sections were stained with hematoxylin for 3–5 min before being sealed with glycerol gelatin. Microscopic examination, image acquisition, and quantitative assessment analysis were performed using ImagePro Plus 6.0 software.

### 2.6 16S rRNA gene sequencing

Following DNA extraction, PCR was conducted with primers designed for conserved regions and sequencing adapters. Illumina Novaseq 6000 system was used for sequencing qualified libraries. High-throughput sequencing data were processed into raw sequencing sequences (Reads) through Base Calling analysis in FASTQ format. Trimmomatic (version 0.33) and Cutadapt (version 1.9.1) were used for quality filtering and primer removal. USEARCH (version 10) overlapped paired-end Reads and filtered data based on length range. Clustering at 97% similarity was performed with a 0.005% OTU threshold. Taxonomic annotation was done using Silva.138 database and OTU clustering at 97% similarity. Alpha and Beta diversity was assessed using QIIME2 and QIIME software. Linear discriminant analysis (LDA) effect size (LEfSe) was applied for differential analysis. A logarithmic LDA score of 4.0 indicated significant differences. A correlation heatmap was used to show the relationship between metabolic indicators and fecal microbiota. Spearman correlation (r) with *p* < 0.05 was used for statistical significance.

### 2.7 Quantitative real-time PCR

Total RNA was extracted from livers using *TransZol* Up (TransGen Biotech, China). The concentration and purity of total RNA were checked. Reverse transcript reaction was done with *Evo M-MLV* RT Premix (Accurate Biology, China). Real-time PCR was performed using SYBR Green Premix *Pro Taq* HS qPCR kit (Accurate Biology, China) on a QuantStudio™ 5 Real-Time PCR Instrument (Applied Biosystems, USA). The mRNA levels of PPARα, CPT1A, SREBP1, FASN, SCD1, ACC1, G6PC, and PCK1 were quantified with β-actin as the normalization control. Primer sequences can be found in Supplementary Table S1.

### 2.8 Western blotting

The liver tissue total protein was extracted using radioimmunoprecipitation assay (RIPA) with 1% protease inhibitor and 1% phosphatase inhibitor, followed by quantification of protein concentration via the BCA assay. Subsequently, the protein samples were separated by SDS-PAGE and transferred onto a PVDF membrane. The membrane was blocked with NcmBlot Blocking Buffer and then incubated overnight at 4°C with the appropriate dilution of primary protein antibodies. The antibodies used included PGRMC1 (1:5000; Proteintech; 12990-1-AP), SIRT1 (1:2000; HUABIO; ER130811), FOXO1 (1:1000; Proteintech; 18592-1-AP), p-FOXO1 (s256) (1:500; ProMab Biotechnologies Inc.; P40793), and β-actin (1:10000; Proteintech; 66009-1-Ig). Subsequently, the corresponding HRP-conjugated Goat Anti-Rabbit (Servicebio Biological, CR2104087) or HRP-conjugated Goat Anti-Mouse (Servicebio Biological, CR2105125) secondary antibody, diluted by 1:3000 with antibody diluent, was added and incubated on a shaker at room temperature for 1 h. Finally, the protein bands were detected using an ECL kit and ChemiDoc Imagers (BIO-RAD, USA). Grayscale values of the Western blot bands were analyzed using the ImageJ software.

### 2.9 Statistical analysis

The experimental data were presented as mean ± standard error of the mean (SEM). GraphPad Prism version 8.0.1 was utilized for data analysis and visualization. The study employed one-way ANOVA with F statistics, followed by Tukey’s multiple comparison test to examine various biochemical parameters, AUC, oil red O staining, pathway mRNA levels, and protein expression across different subgroups. Furthermore, GTT, ITT, and body weight data were subjected to two-way ANOVA with F statistics, followed by Tukey’s multiple comparison test. Statistical significance was set at *p* < 0.05.

## 3 Results

### 3.1 Effects of AKK on OLZ-induced weight gain and hepatic injury in mice

To investigate the effects of AKK on OLZ-induced metabolic dysfunction in mice, we evaluated alterations in body weight and hepatic mass. As the feeding time prolonged, the weight of mice in each group increased to varying degree (Figure 1c). Compared with NC mice, HFD diet notaly induced weight gain in the late stage of treatment. In HFD mice, OLZ did not induce further weight gain despite increasing the ratio of liver weight to body weight (Figure 1d). In contrast, AKK supplementation decreased body weight in the late stage of treatment compared to mice receiving OLZ.

We next evaluated the effects of AKK on OLZ-induced hepatic injury in mice. Interestingly, OLZ treatment manifested signs of hepatic injury with increased levels of serum ALT (F_4, 40_ = 6.429, *p* = 0.0004) and AST (F_4, 40_ = 5.149, *p* = 0.0019) in mice that were reversed by AKK co-administration (Figures 1e, f). In addition, AKK supplementation also restored hepatic oxidative stress induced by OLZ with decreased hepatic TNF-α (F_4, 30_ = 18.79, *p* < 0.0001) and MDA (F_4, 35_ = 16.67, *p* < 0.0001) levels (Figures 1g, h). Hepatic H&E staining results exhibited notable histological changes especially hepatic steatosis characterized as widely distributed lipid accumulation in mice with HFD diet only (Figure 1b). Compared with NC mice. Interestingly, OLZ treatment aggravated this phenomenon which was mitigated by AKK supplementation.

### 3.2 AKK protects against OLZ-induced disturbance of lipid metabolism

**FIGURE 2:**
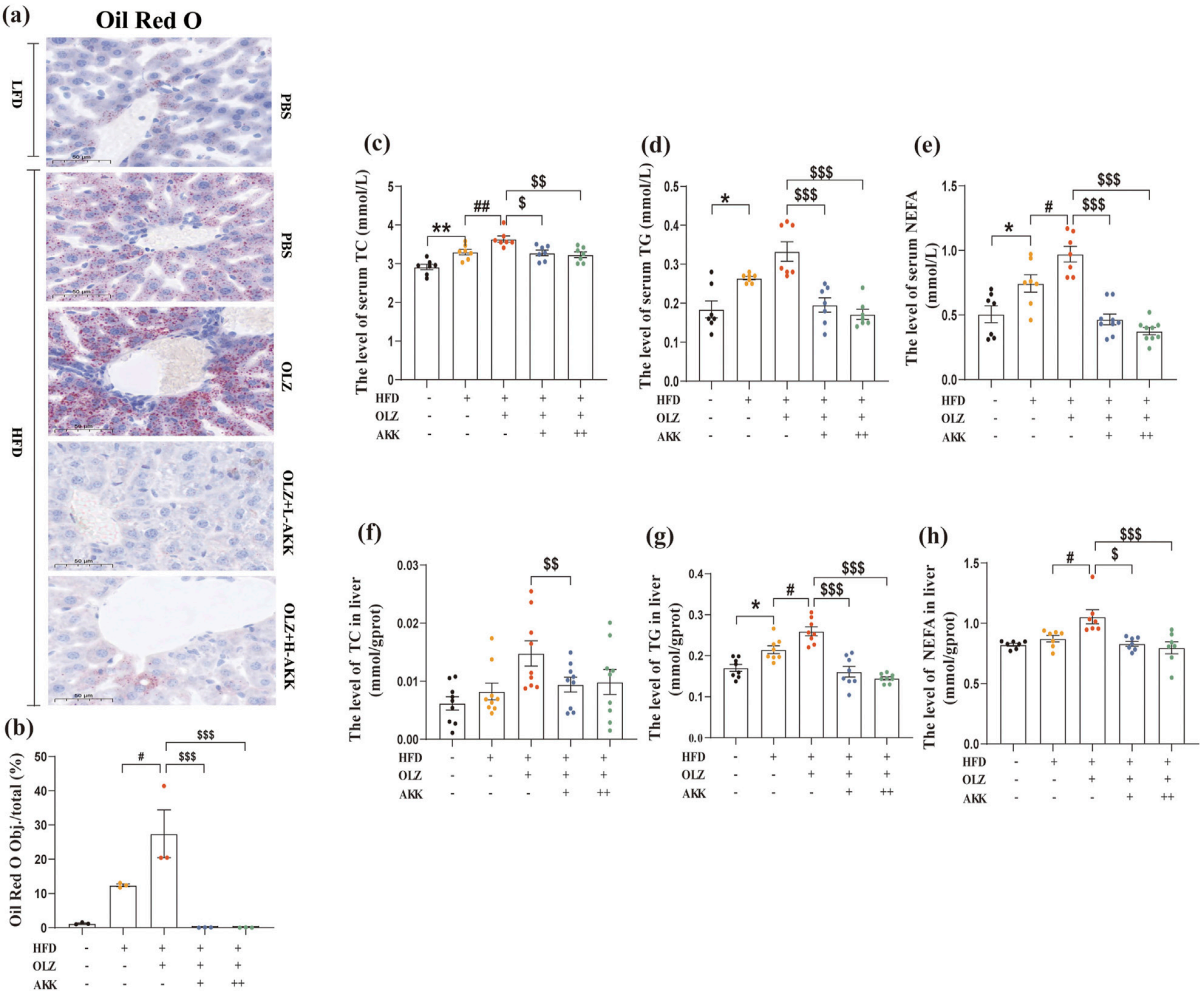
Effects of AKK on OLZ-induced disturbance of lipid metabolism. **(a)** Oil Red O staining (scale bar = 50 μm). **(b)** Statistical analysis of ORO staining. **(c)** The level of serum TC. **(d)** The level of serum TG. **(e)** The level of serum NEFA. **(f)** The level of TC in liver. **(g)** The level of TG in liver. **(h)** The level of NEFA in liver. Differences were considered statistically significant when *p* < 0.05. ^*^ *p* < 0.05, ^**^ *p* < 0.01, ^***^ *p* < 0.001, HFD vs. LFD. ^#^ *p* < 0.05, ^##^ *p* < 0.01, ^###^ *p* < 0.001, OLZ vs. HFD. ^$^ *p* < 0.05, ^$$^ *p* < 0.01, ^$$$^ *p* < 0.001, OLZ-AKK vs. OLZ-PBS.

Next, we investigated the effect of AKK on lipid metabolism in OLZ-treated mice. ORO staining was used to detect the distribution of triglyceride in liver tissues. OLZ aggravated hepatic lipid accumulation in HFD mice (Figures 2a, b) whereas AKK supplementation mitigated this phenomenon (F_4, 10_ = 12.96, *p* = 0.0006). In comparison with NC mice, HFD diet significantly elevated serum TC (F_4, 30_ = 12.08, *p* < 0.0001), TG (F_4, 30_ = 14.13, *p* < 0.0001) and NEFA (F_4, 34_ = 21.76, *p* < 0.0001) levels that was further exacerbated by OLZ treatment. Inspiringly, AKK supplementation significantly reduced serum TC, TG and NEFA levels (Figures 2c–e). Similar tendencies for TC (F_4, 35_ = 3.813, *p* = 0.0113), TG (F_4, 35_ = 23.64, *p* < 0.0001) and NEFA (F_4, 30_ = 7.445, *p* = 0.0003) were also observed in liver tissues (Figures 2f–h).

### 3.3 AKK protects against OLZ-induced disturbance of glucose metabolism

**FIGURE 3:**
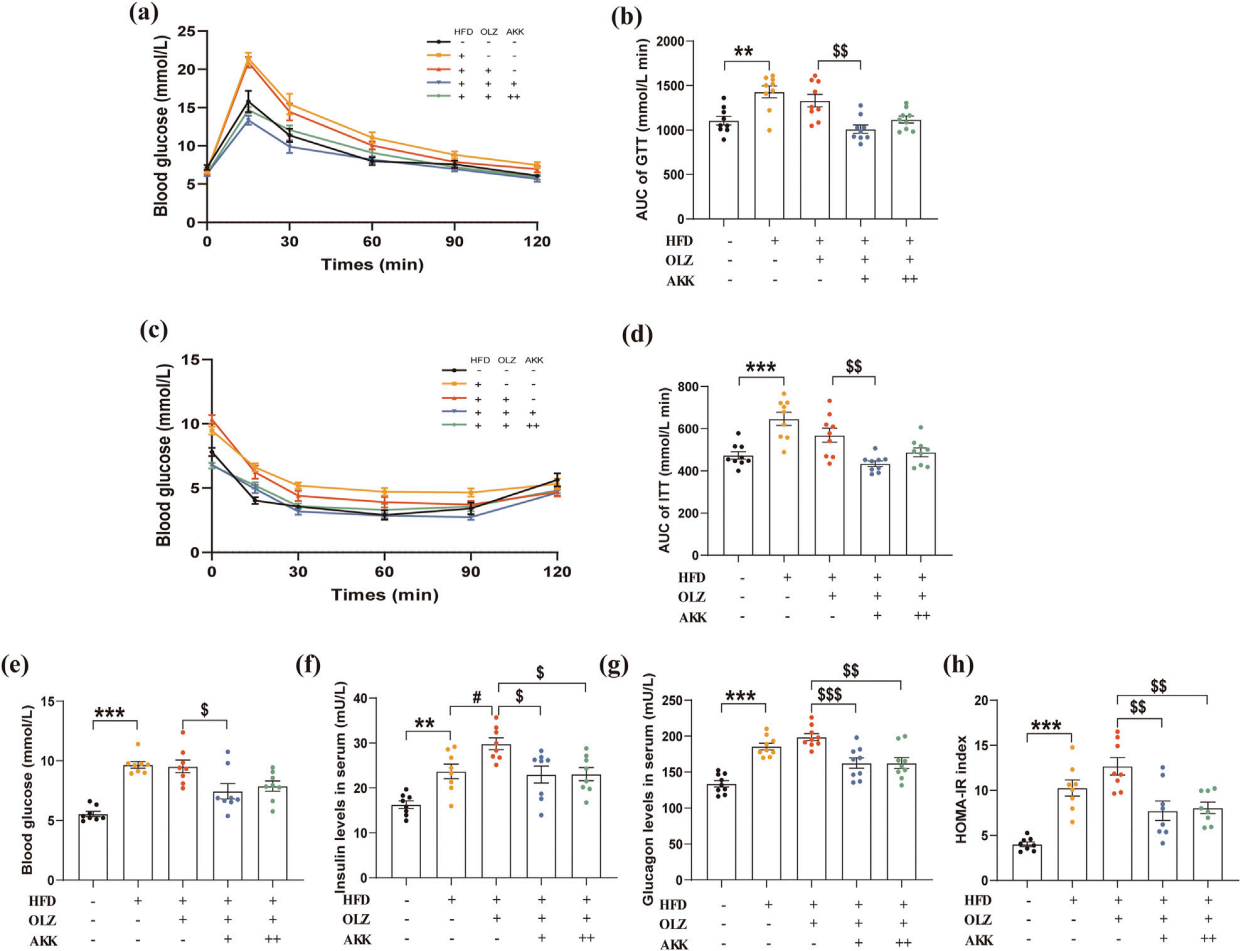
Effects of AKK on OLZ-induced disturbance of glucose metabolism. **(a)** GTT data for mice. **(b)** Area under curve for IGTT. **(c)** ITT data for mice. **(d)** Area under curve for ITT. **(e)** Fasting blood glucose level. **(f)** Serum insulin level. **(g)** Serum glucagon level. **(h)** HOMA-IR index data. Differences were considered statistically significant when *p* < 0.05. ^*^ *p* < 0.05, ^**^ *p* < 0.01, ^***^ *p* < 0.001, HFD vs. LFD. ^#^ *p* < 0.05, ^##^ *p* < 0.01, ^###^ *p* < 0.001, OLZ vs. HFD. ^$^ *p* < 0.05, ^$$^ *p* < 0.01, ^$$$^ *p* < 0.001, OLZ-AKK vs. OLZ-PBS.

Next, we evaluated the effect of AKK on glucose metabolism in OLZ-treated mice. During the GTT test, the AUC of GTT in the HFD group was significantly higher than that in the NC group, suggesting impairment of glucose tolerance (Figures 3a, b). There was no further impairment of glucose tolerance by OLZ treatment in the HFD group and AKK supplementation in low dosage improved glucose intolerance in comparison with OLZ group. During the ITT test, OLZ treatment did not aggravate insulin resistance induced by HFD. Likewise, AKK coadministration in low dosage improved insulin resistance in OLZ-treated mice (Figures 2c–d). In addition, we also detected parameters related with glucose metabolism in fasting conditions. In comparison with NC mice, HFD significantly elevated the levels of fasting blood glucose (F_4, 35_ = 14.25, *p* < 0.0001), serum insulin (F_4, 35_ = 10.73, *p* < 0.0001) and serum glucagon (F_4, 40_ = 17.47, *p* < 0.0001) (Figures 3e–g). Consistent with ITT results, HOMA-IR (F_4, 35_ = 15.31, *p* < 0.0001) directly indicated the occurrence of insulin resistance in HFD group, which was mitigated by AKK supplementation (Figure 3h).

### 3.4 AKK restored the effects of OLZ-induced disturbance in lipid synthesis, oxidation and gluconeogenesis

**FIGURE 4:**
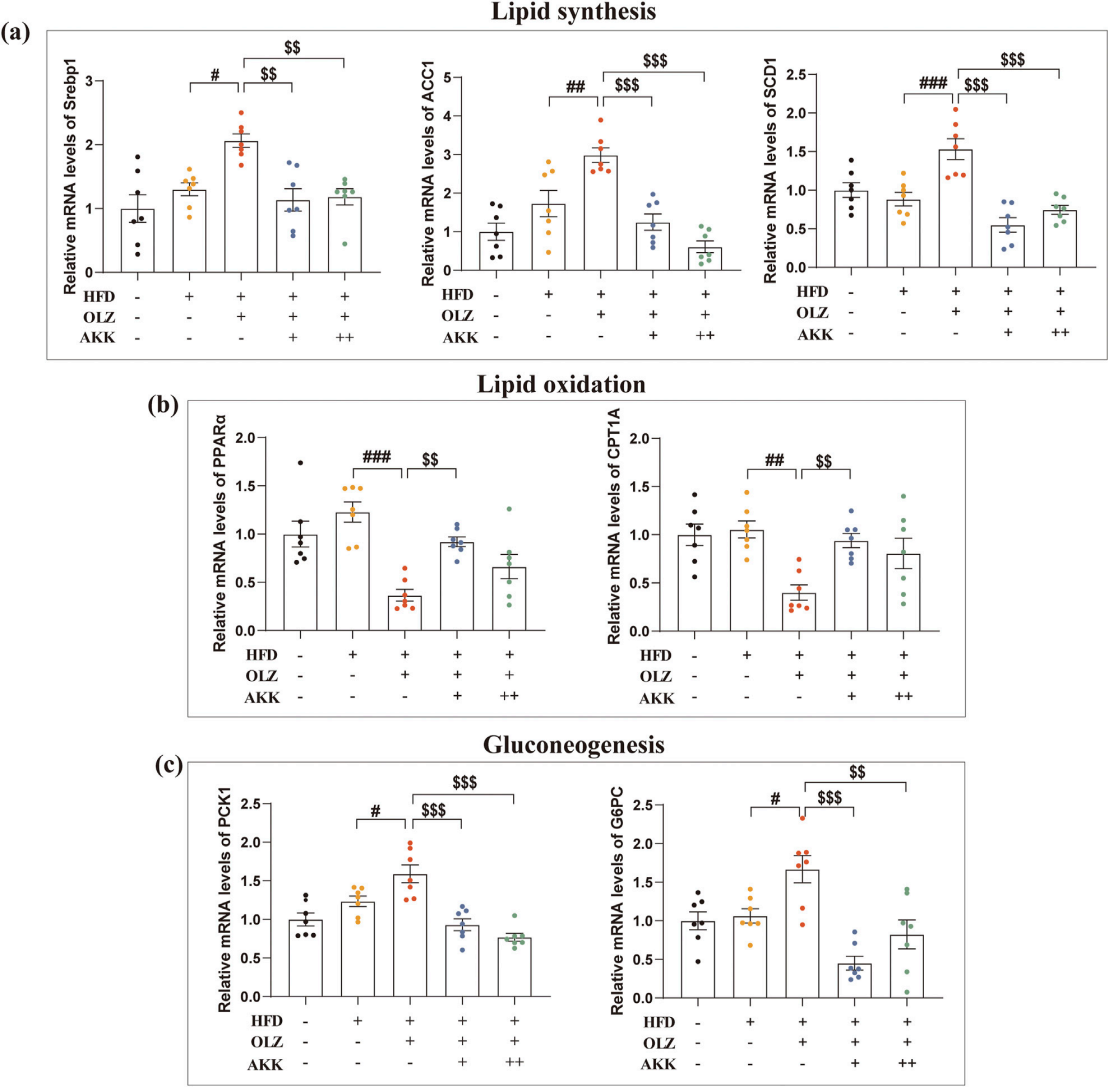
Effects of AKK on OLZ-induced disturbance in lipid synthesis, oxidation and gluconeogenesis. **(a)** Relative mRNA levels of Srebp1, ACC1 and SCD1in lipid synthesis. **(b)** Relative mRNA levels of PPARα and CPT1A in lipid oxidation. **(c)** Relative mRNA levels of PCK1 and G6PC in gluconeogenesis. Differences were considered statistically significant when *p* < 0.05. ^*^ *p* < 0.05, ^**^ *p* < 0.01, ^***^ *p* < 0.001, HFD vs. LFD. ^#^ *p* < 0.05, ^##^ *p* < 0.01, ^###^ *p* < 0.001, OLZ vs. HFD. ^$^ *p* < 0.05, ^$$^ *p* < 0.01, ^$$$^ *p* < 0.001, OLZ-AKK vs. OLZ-PBS.

To explore the mechanism by which AKK improves OLZ-related metabolic dysfunction, we examined the mRNA levels of key enzymes in hepatic lipid synthesis, lipid oxidation and gluconeogenesis. Interestingly, no significant differences were observed between HFD and NC group. In HFD condition, OLZ treatment notably enhanced lipid synthesis by upregulating the mRNA expression of Srebp1 (F_4, 30_ = 7.661, *p* = 0.0002), ACC1 (F_4, 30_ = 15.61, *p* < 0.0001) and SCD1 (F_4, 30_ = 14.63, *p* < 0.0001) as shown in Figure 4a. Meanwhile, OLZ treatment inhibited lipid oxidation by downregulating PPARα (F_4, 30_ = 10.72, *p* < 0.0001) and CPT1A (F_4, 30_ = 6.053, *p* = 0.0011) mRNA expression (Figure 4b). AKK supplementation attenuated the expression of factors above involved in lipid metabolism. Additionally, OLZ-treated mice had higher hepatic levels of PCK1 (F_4, 30_ = 15.20, *p* < 0.0001) and G6PC (F_4, 30_ = 10.16, *p* < 0.0001) compared to HFD mice but a return to the baseline level was observed upon AKK co-administration (Figure 4c).

### 3.5 Effects of AKK on OLZ-induced disturbance in gut microbiota

**FIGURE 5:**
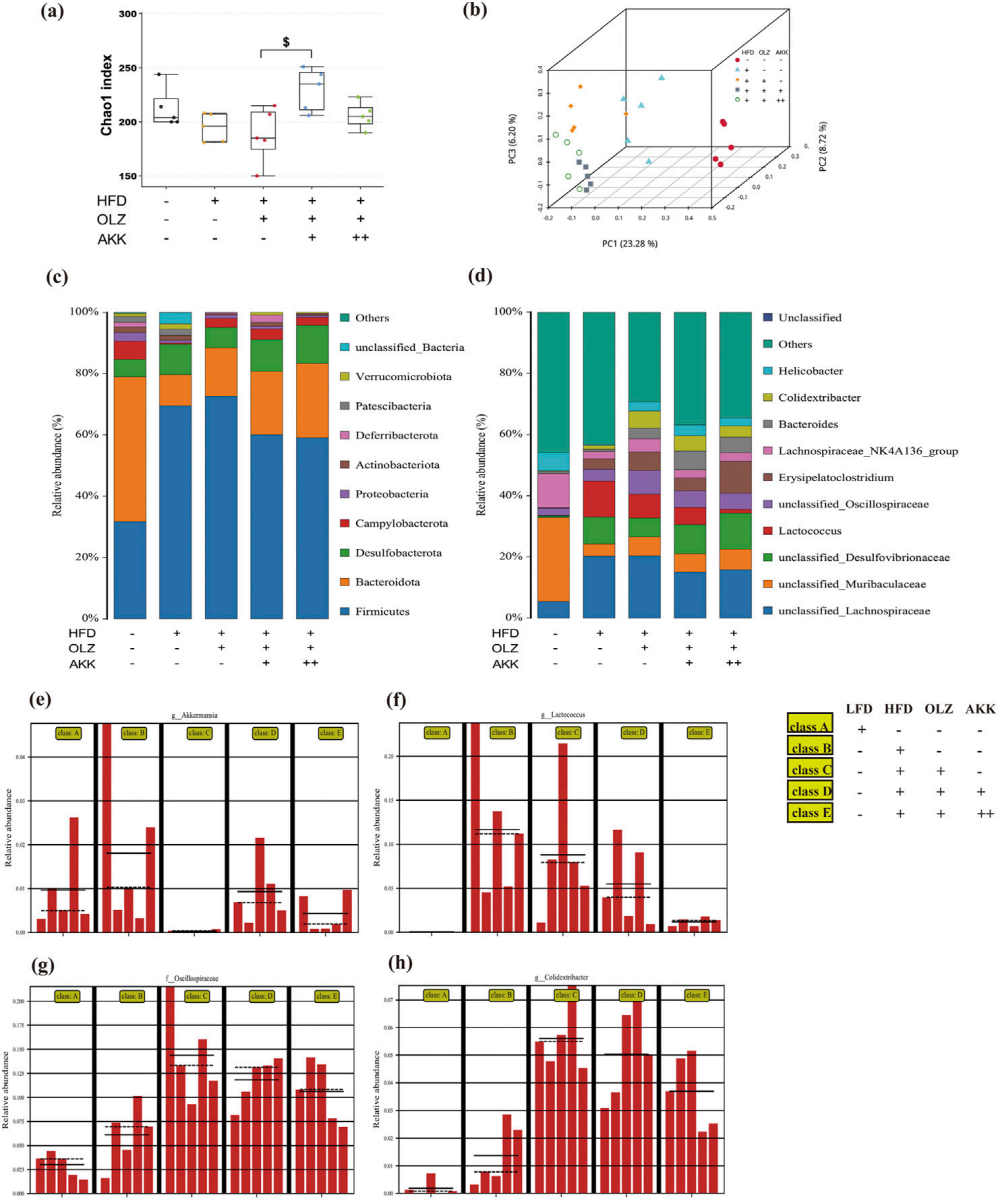
Effects of AKK on OLZ-induced disturbance in gut microbiota. **(a)** Bacterial community richness (measured by the Chao1 index). **(b)** Principal coordinates analysis. Percentages of community abundance on phylum **(c)** and genus levels **(d)**. Relative abundance of AKK **(e)**, *Lactococcus* **(f)**, *Oscillospiraceae* **(g)** and *Colidextribacter* **(h)**. Differences were considered statistically significant when *p* < 0.05 (n = 5 for each group).

In order to explore the role of gut microbiota in OLZ-induced metabolic disorders, we analyzed the changes with high-throughput sequencing using bacterial 16S rRNA. The alpha diversity observed for the gut microbiota in the five groups was assessed using the richness index (Chao1). As indicated in Figure 5a, the NC group’s gut samples showed higher estimates of richness and diversity than the HFD group. However, no significant differences were observed between HFD and OLZ-treated group. Inspiringly, AKK co-administration (low dosage) markedly increased the chao1 index. Additionally, the beta diversity of the gut microbiota was evaluated using principal coordinates analysis (PCoA). The PCoA diagram showed a clear distinction among the five groups, with the first, second and third principal coordinates providing 23.28%, 8.72% and 6.20% of the total variation, respectively (Figure 5b).

The composition of the gut microbial community was categorized at the phylum and genus levels. The dominant phyla included Fusobacteria, Bacteroidetes and Desulfobacterota in all groups (Figure 5c). The Fusobacteria phylum was clearly dominant in the HFD and OLZ groups. However, Bacteroidetes predominated in the NC group. When considering the effects at the phylum level, HFD and OLZ exposure increased the ratio of Firmicutes/Bacteroidetes while AKK co-administration lowered the ratio. At the genus level, unclassified_Lachnospiraceae and Lactococcus were clearly dominant in the HFD and OLZ groups. In comparison with OLZ group, AKK co-administration increased the relative richness of unclassified_Desulfovibrionaceae and Erysipelatoclostridium with a decrease in the abundance of Lactococcus (Figure 5d).

To further analyze the changes in intestinal microbial species abundance, the LEfSe method was used to identify species with statistical differences between groups. Notably, four bacterial genera, namely *A.muciniphila* ^*sub*^, *Lactococcus*, *Oscillospiraceae*, and *Colidextribacter*, demonstrated significant variations across all groups (Figures 5e–h). The analysis revealed a marked decrease in the relative abundance of AKK in the OLZ group compared to the HFD group, whereas the relative abundances of *Oscillospiraceae* and *Colidextribacter* were significantly elevated. Furthermore, *Lactococcus* predominated in both the HFD and OLZ groups. Supplementation with AKK ameliorated the dysbiosis of the intestinal microbiota, evidenced by the reduced relative abundance of *Lactococcus*, *Oscillospiraceae*, and *Colidextribacter*, alongside an increased relative abundance of *A.muciniphila* ^*sub*^. These findings imply that co-administration of AKK not only facilitates the colonization of AKK but also mitigates the imbalance of other bacterial genera induced by OLZ treatment.

### 3.6 Correlations between lipid and glucose metabolism-related markers and microbial abundance

**FIGURE 6:**
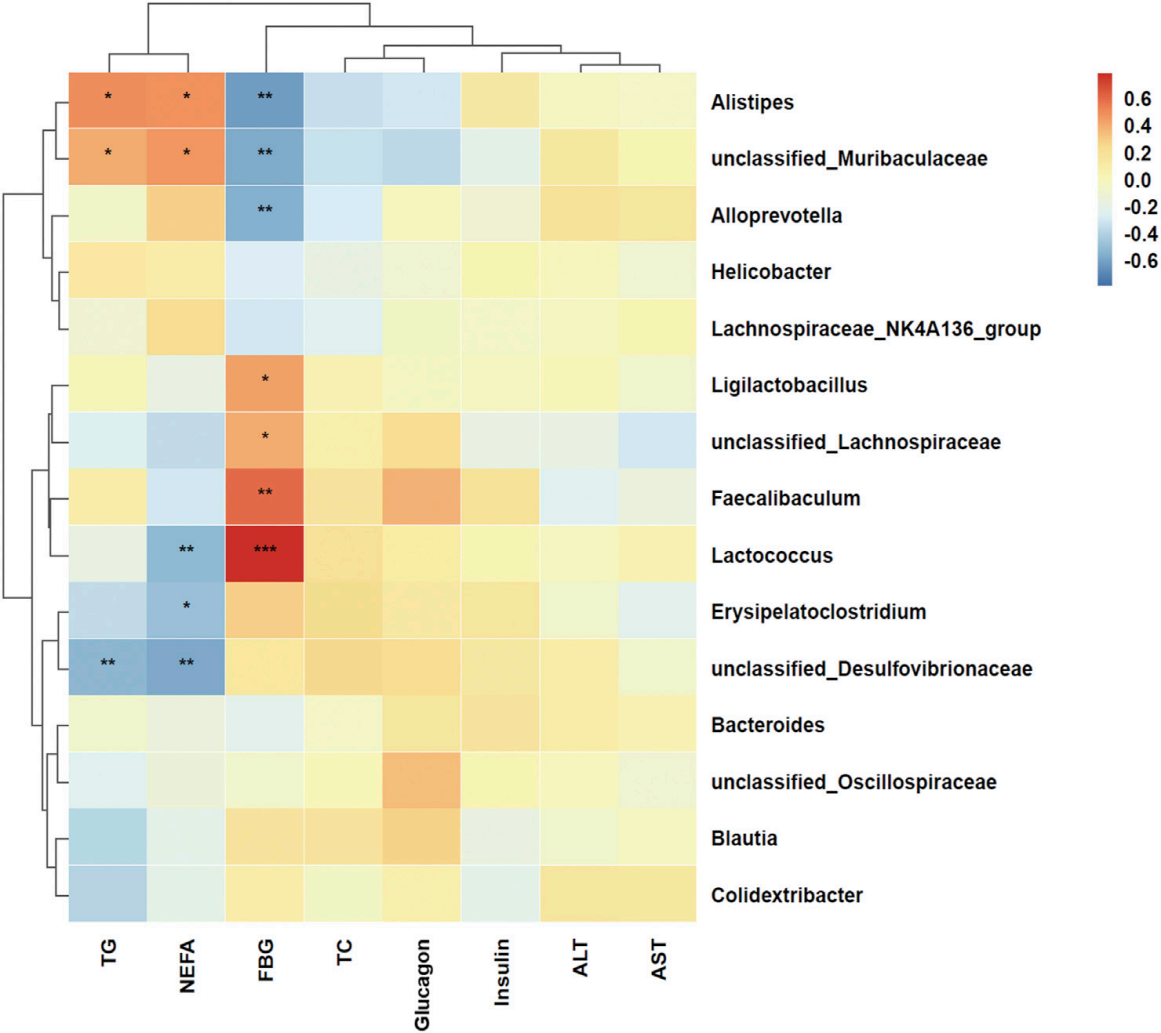
Heat map of spearman correlation between lipid and glucose metabolism indicators and genus level of gut microbiota. Red represents a positive correlation and blue represents a negative correlation. ^*^ *p* < 0.05, ^**^ *p* < 0.01, ^***^ *p* < 0.001.

To visualize the correlation between the gut microbiome and serum lipid and glucose metabolism-related indicators, p-values were calculated using by Spearman algorithm. At the genus level (Figure 6), *Alistipes* (*p* = 0.012) and *unclassified_Muribaculaceae* (*p* = 0.042) were positively correlated with serum TG, whereas *unclassified_Desulfovibrionaceae* (*p* = 0.008) was negatively correlated with serum TG. *Alistipes* (*p* = 0.014) and *unclassified_Muribaculaceae* (*p* = 0.017) were positively associated with serum NEFA. *Lactococcus* (*p* = 0.009), *Erysipelatoclostridium* (*p* = 0.013) and *unclassified_ Desulfovibrionaceae* (*p* = 0.003) were negatively associated with serum NEFA. *Alistipes* (*p* = 0.001), *unclassified_ Muribaculaceae* (*p* = 0.003) and *Alloprevotella* (*p* = 0.004) were negatively related with fasting blood glucose. *Ligilactobacillus* (*p* = 0.028), *unclassified_Lachnospiraceae* (*p* = 0.043), *Faecalibaculum* (*p* = 0.001) and *Lactococcus* (*p* < 0.001) were positively related with fasting blood glucose. Overall, these results demonstrate that OLZ administration induces significant remodeling of the gut microbiota, leading to metabolic disturbances. This microbial alteration likely contributes to the observed disruptions in lipid and glucose metabolism associated with OLZ exposure.

### 3.7 Inhibitory effects on hepatic PGRMC1/SIRT1/FOXO1 signaling pathway by olanzapine and the reversal effects of AKK

**FIGURE 7:**
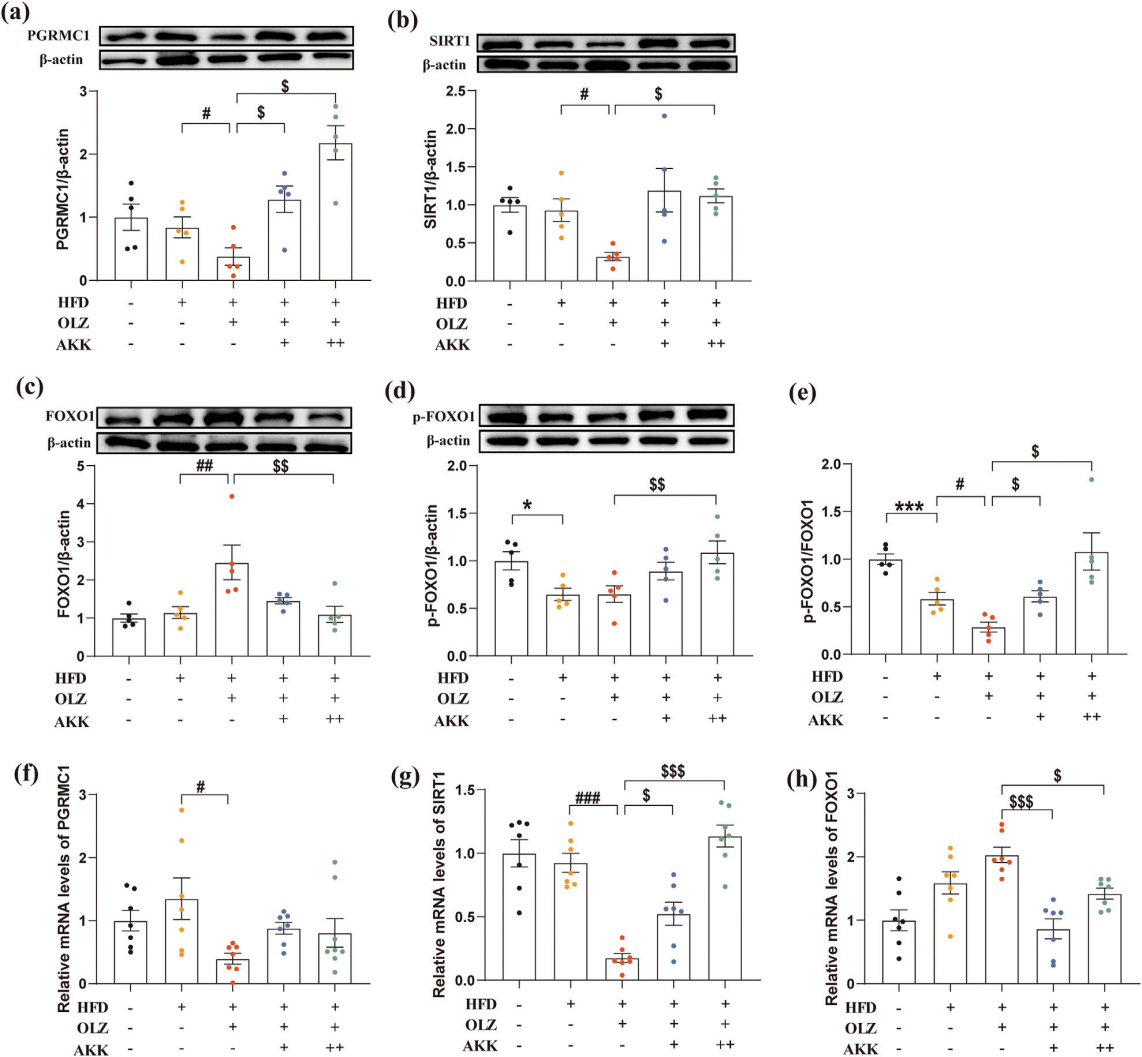
Effects of AKK and OLZ on the hepatic PGRMC1/SIRT1/FOXO1 signaling pathway. Relative mRNA expression of PGRMC1 **(a)**, SIRT1 **(b)**, FOXO1 **(c)**. Relative protein expression of PGRMC1 **(d)**, SIRT1 **(e)**, FOXO1 **(f)**, p-FOXO1 **(g)**. **(h)** The ratio of p-FOXO1/FOXO1. Differences were considered statistically significant when *p* < 0.05. ^*^ *p* < 0.05, ^**^ *p* < 0.01, ^***^ *p* < 0.001, HFD vs. LFD. ^#^ *p* < 0.05, ^##^ *p* < 0.01, ^###^ *p* < 0.001, OLZ vs. HFD. ^$^ *p* < 0.05, ^$$^ *p* < 0.01, ^$$$^ *p* < 0.001, OLZ-AKK vs. OLZ-PBS.

Given the crucial role of PGRMC1 and its downstream SIRT1/FOXO1 in hepatic lipid and glucose metabolism, the hepatic expression of these key proteins was examined in mice. As indicated in Figures 7a–e, compared to the NC group, a significant decrease in the ratio of p-FOXO1 to FOXO1 was observed in HFD mice (*p* = 0.0006). Under high-fat diet conditions, OLZ exposure markedly reduced the expression of the PGRMC1 (*p* = 0.0300), SIRT1 (*p* = 0.0208), and the ratio of p-FOXO1 to FOXO1 (*p* = 0.0136). When combined with AKK administration, the protein expression of PGRMC1(L: *p* = 0.0333; H: *p* = 0.0198) and the ratio of p-FOXO1 to FOXO1 (L: *p* = 0.0222; H: *p* = 0.0339) returned to baseline levels while only the high dosage of AKK administration restored the SITR1 expression (*p* = 0.0137).

Additionally, we also measured the mRNA expression of PGRMC1/SIRT1/FOXO1 signaling pathway. As shown in Figures 7f–h, OLZ exposure reduced the mRNA levels of PGRMC1 (F_4, 31_ = 2.784, *p* = 0.0438) and SIRT1 (F_4, 30_ = 22.82, *p* < 0.0001) in mice under high-fat diet conditions. AKK co-administration significantly increased the mRNA level of SIRT1, while the mRNA level of FOXO1 (F_4, 30_ = 10.57, *p* < 0.0001) was significantly decreased. Thess results showed that AKK can reverse the abnormal mRNA expression of PGRMC1/SIRT1/FOXO1 pathway induced by OLZ to a certain extent.

## 4 Discussion

**FIGURE 8:**
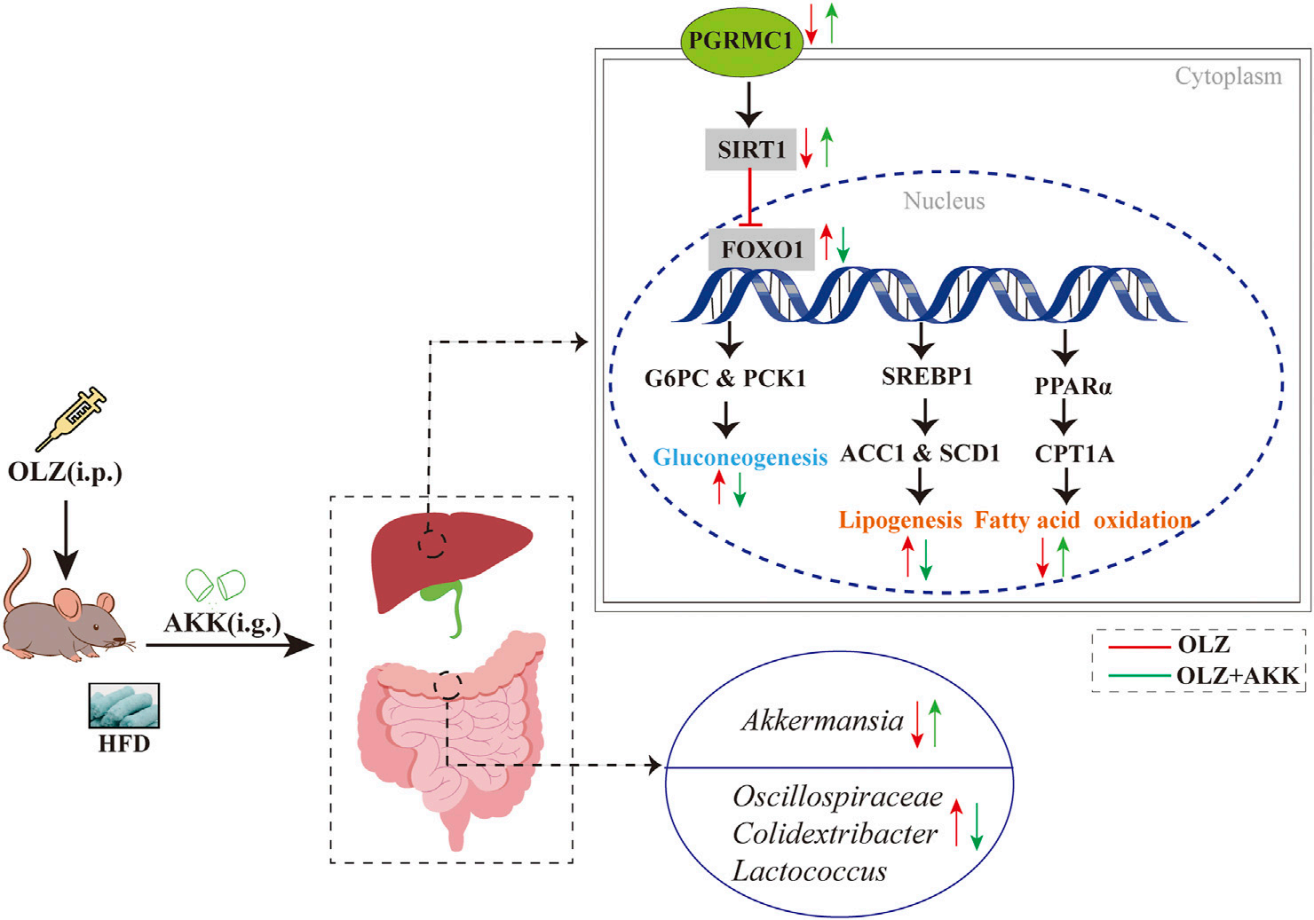
A potential mechanism underlying attenuation of OLZ-induced metabolic dysfunction-associated steatotic liver disease through enriching AKK in mice.

In the study, a well-established mouse model of MASLD was induced by chronic treatment with OLZ and HFD. Furthermore, OLZ exposure under HFD conditions led to significant hepatic lipid accumulation and impaired glucose metabolism, along with disruptions in the gut microbiota. Notably, AKK ameliorated gut dysbiosis, counteracting OLZ-induced lipid and glucose metabolic disturbances, potentially through the PGRMC1/SIRT/FOXO1 pathway (Figure 8). These findings suggest that AKK supplementation may represent a promising therapeutic strategy for the treatment of OLZ-induced MASLD.

Our study confirmed that OLZ-induced hepatic injury is independent of weight gain under HFD condition. As stated, rodents with the low doses of OLZ appear to be more effective in weight gain than those with high doses of OLZ (Arjona et al., 2004; Zhu et al., 2023). However, such low doses lack clinical relevancies. The MASLD model induced by OLZ and HFD diet was mainly manifested as enhanced hepatic oxidative stress, hepatic dyslipidemia, and insulin resistance, all of which contribute to the development of MASLD (Nimura et al., 2015; Chen et al., 2017; Li et al., 2019). The gut microbiota plays a critical role in regulating host metabolism, and its dysregulation has been implicated in the pathogenesis of MASLD (Drożdż et al., 2021). OLZ-induced metabolic disturbances are thought to be partly mediated by alterations in gut microbiota composition and function (Morgan et al., 2014; Kao et al., 2018). In our study, OLZ led to significant gut dysbiosis, manifested as an increase in pathogenic bacteria associated with metabolic dysfunction and a decrease in beneficial bacteria, such as AKK. Classified as “lean bacteria”, AKK is now recognized as a promising candidate for improving metabolic disorders, including obesity, diabetes, liver diseases, and cardiometabolic conditions (Cani and Vos, 2017). Recent studies have highlighted the effectiveness of AKK in combating MASLD through reshaping the gut microbiome to restore a favorable balance between beneficial and harmful bacteria. Specifically, OLZ reduced the relative abundance of beneficial bacteria while increasing pathogenic bacteria. In our study, AKK administration reduced the levels of pathogenic bacteria such as *Lactococcus*, *Oscillospiraceae*, and *Colidextribacter*, contributing to the improvement of gut microbial health.

*Lactococcus* is a key microbial group in obesity and fatty liver disease (Singh et al., 2017; Jo et al., 2021; Xu et al., 2021). Studies have shown that the abundance of *Lactococcus* increases in mice fed a high-fat diet, and its relative abundance is positively correlated with serum insulin, LPS, TC, and liver TG levels (M. Wang et al., 2022). *Lactococcus* belongs to the lactic acid bacteria family, which primarily converts lactose from dairy products into lactic acid, a function that also applies to the human gut (Vos et al., 2005). The GPR81 lactate receptor is activated by lactic acid produced by gut microbiota, influencing fat breakdown and potentially leading to lipid metabolism disorders (Husted et al., 2017). Besides, lactic acid has been reported to accumulate as MASLD progresses (Scheiner et al., 2017; Luo et al., 2023) and in patients following antipsychotic treatment (Elmorsy et al., 2016). These findings suggest that the interaction between the microbiome and glucose-lipid metabolism might be mediated by gut microbiome-derived metabolites such as lactic acid. Moreover, *Oscillibacter* and *Colidextribacter* have been identified as inflammation-associated gut microbes and are highly enriched in mouse models of colitis (Lan et al., 2023; Yang et al., 2023). Consistent with our study, the relative abundance of *Oscillibacter* and *Colidextribacter* was enriched in OLZ group, accompanied with elevated TNF-α and MDA levels. Another study also reported a positive correlation between the relative abundance of *Colidextribacter* and serum MDA levels (Chen et al., 2022). At the phylum level, OLZ significantly increased Firmicutes-to-Bacteroidetes ratio, while intervention with AKK effectively reversed this effect. The Firmicutes-to-Bacteroidetes ratio is a key factor in the development of MASLD. Studies have shown that a higher Firmicutes-to-Bacteroidetes ratio is positively correlated with hepatic steatosis in obese patients with MASLD (Jasirwan et al., 2021). This correlation suggests that dysbiosis at the phylum level may contribute to the development of liver diseases, such as MASLD, by altering hepatic metabolism and inflammatory responses (Wang et al., 2016).

To further elucidate the potential therapeutic mechanism by which AKK supplementation mitigates OLZ-induced MASLD, PGRMC1 was identified as a key target in our study. Our experiments showed that OLZ binds strongly to PGRMC1, as confirmed by surface plasmon resonance (SPR) and molecular docking assays (Zeng et al., 2024). The binding of OLZ to PGRMC1 may be attributable to multiple adverse effects of the drugs including increased lipid biosynthesis (Zhu et al., 2023) and disturbed glucose metabolism (Cao et al., 2021). Notably, we found that AKK supplementation upregulated hepatic PGRMC1 expression, which was accompanied by reductions in ALT, AST, MDA, and TNF-α levels, suggesting that PGRMC1 plays a role in mediating AKK’s therapeutic effects.

Current evidence strongly supports the role of gut microbiota in regulating the PGRMC1/SIRT1/FOXO1 pathway. First, prebiotic B-GOS has been shown to alleviate OLZ-induced lipid metabolism disorders by increasing the abundance of AKK and counteracting OLZ’s negative effects on the PGRMC1 pathway (Zeng et al., 2024). Second, apple polyphenols have been found to mitigate hepatic steatosis by promoting AKK abundance, an effect that depends on the activation of the SIRT1 pathway (Yin et al., 2022). Additionally, symbiotic gut bacteria and intestinal epithelial cells interact through the IR/IGF1R-FOXO1 pathway, playing a critical role in metabolic regulation (Ostermann et al., 2019). In our study, AKK effectively reversed OLZ-induced dysregulation of the hepatic PGRMC1/SIRT1/FOXO1 pathway, which may explain its impact on downstream genes involved in lipid synthesis, oxidation, and gluconeogenesis.

FOXO1 activity plays a crucial role in the regulation of key genes involved in glucose and lipid metabolism, with dysregulation contributing to the development of MASLD (Ramadan et al., 2022). Specifically, overexpression of FOXO1 leads to upregulation of genes such as SREBP-1c, FAS, ACC, and SCD1, which promote triglyceride synthesis (Gao et al., 2022). When insulin signaling is impaired, FOXO1 activity increases, driving the expression of G6PC and PCK1 genes, resulting in excessive glucose production. Additionally, FOXO1 enhances Akt signaling and inhibits PPARα expression, thereby reducing fatty acid oxidation (Matsumoto et al., 2006). Studies have demonstrated that reduced SIRT1 levels decrease FOXO1 phosphorylation, which in turn upregulates fatty acid synthesis genes and slows fatty acid β-oxidation (Sun et al., 2013). Our study provides further evidence that AKK significantly improves insulin resistance and lipid disturbances, thereby reducing fat accumulation and lowering the risk of type 2 diabetes. In line with our findings, Huang et al. reported that AKK significantly decreased the expression of G6Pase and PEPCK, leading to reduced blood glucose levels (Huang et al., 2021).

Overall, AKK shows promising potential to lower blood lipid levels, exhibit anti-inflammatory effects, and improve insulin resistance. However, although AKK has beneficial effects on metabolism, excessive levels may disrupt mucin breakdown, weaken the intestinal barrier, and trigger inflammation (e.g., IL-1β, IL-6, TNF-α) (Chiantera et al., 2023). In a mouse model of intestinal neoplasia, AKK gavage may promote colorectal cancer development by increasing tumor number and size (Wang et al., 2022). Moreover, its role in mitigating OLZ-induced MASLD remains insufficiently explored, especially the main material basis of AKK that regulate lipid metabolism is far from clear. Current evidence suggests that metabolic disturbances induced by atypical antipsychotic drugs (AAPDs) are closely linked to changes in gut microbiome composition and alterations in microbial metabolites, such as short-chain fatty acids (SCFAs) (Foster and McVey Neufeld, 2013). Future research should employ multi-omics techniques to identify the specific genes/proteins/metabolites mediating the therapeutic effects of AKK in MASLD models. Additionally, the development of a drug-microbiome interaction model could evaluate how different microbiota affect OLZ metabolism and efficacy, providing a theoretical foundation for personalized treatment strategies. Further research is also needed to clarify the application fields and describe the safety of AKK.

## Data Availability

The raw 16S rRNA sequencing data presented in the study are deposited in the Sequence Read Archive repository, accession number PRJNA1189011, available at: https://www.ncbi.nlm.nih.gov/bioproject/PRJNA1189011.
