# Supplementary material for: Akkermansia muciniphila ameliorates olanzapine-induced metabolic dysfunction-associated steatotic liver disease via PGRMC1/SIRT1/FOXO1 signaling pathway
Source: Front Pharmacol. 2025 Mar 19;16:1550015. doi: 10.3389/fphar.2025.1550015 (PMC11961884; doi:10.3389/fphar.2025.1550015)
Supplement: Supplementary file 1 [file Table1.docx]

**Supplementary Table S1. Primer sequences.**

| Gene | Forward primer (5' to 3') | Reverse primer (5' to 3') |
| --- | --- | --- |
| β-actin | GGCTGTATTCCCCTCCATCG | CCAGTTGGTAACAATGCCATGT |
| PPARα | AGGCTGTAAGGGCTTCTTTC | GCATTTGTTCCGGTTCTTCTTC |
| CPT1A | CTATGCGCTACTCGCTGAAGG | GGCTTTCGACCCGAGAAGA |
| SREBP1 | CGGAACCATCTTGGCAACAGT | CGCTTCTCAATGGCGTTGT |
| FASN | GGAGGTGGTGATAGCCGGTAT | TGGGTAATCCATAGAGCCCAG |
| SCD1 | TTCTTGCGATACACTCTGGTGC | CGGGATTGAATGTTCTTGTCGT |
| ACC1 | GTTTCTCTGGTGGGATGAAAGA | GGGCGGGATGTAAACCATTA |
| G6PC | CGGGGCATCTACAATGCCAG | GGCTGGCAAAGGGTGTAGTG |
| PCK1 | GAGATAGCGGCACAAT | TTCAGAGACTATGCGGTG |
